# Association of Polymorphisms in the Glutathione S-Transferase Theta-1 Gene with Cirrhosis and Hepatocellular Carcinoma in Brazilian Patients with Chronic Hepatitis C

**DOI:** 10.3390/vaccines9080831

**Published:** 2021-07-29

**Authors:** Oscar C. Araujo, Vanessa S. de Paula, Kycia M. do Ó, Cristiane A. Villela-Nogueira, Natalia M. Araujo

**Affiliations:** 1 Laboratory of Molecular Virology, Oswaldo Cruz Institute, FIOCRUZ, Rio de Janeiro 21040-360, Brazil; araujo.orc@gmail.com (O.C.A.); vdepaula@ioc.fiocruz.br (V.S.d.P.); 2 São Lucas Hospital, Petrópolis 25660-290, Brazil; kyciadoo@gmail.com; 3 Hepatology Division, Clementino Fraga Filho University Hospital, School of Medicine, Federal University of Rio de Janeiro, Rio de Janeiro 21941-617, Brazil; crisvillelanog@gmail.com

**Keywords:** hepatitis C virus, glutathione S-transferases, cirrhosis, hepatocellular carcinoma, polymorphisms

## Abstract

Oxidative stress contributes to hepatitis C virus (HCV)–induced liver damage. Host genetic factors may be involved in progression of HCV infection. The present study was conducted to determine the influence of glutathione S-transferase (GST)-M1 and T1 gene polymorphisms during different stages of HCV infection, including chronic hepatitis, cirrhosis, and hepatocellular carcinoma (HCC). The study population comprised 190 patients (47 with chronic hepatitis, 83 with cirrhosis (without HCC), and 60 with HCC). *GSTM1* and *GSTT1* gene polymorphisms were analyzed via multiplex polymerase chain reaction. The *GSTT1*-null genotype was more commonly detected in patients with cirrhosis (*n* = 17; 20.5%) and HCC (*n* = 13; 21.7%) than those with chronic hepatitis (*n* = 3; 6.4%). The differences in *GSTT1*-null genotype frequencies were significant for cirrhosis vs. chronic hepatitis (odds ratio, OR, 3.778 (95% confidence interval, CI, 1.045–13.659); *p* = 0.043) and HCC vs. chronic hepatitis (OR, 4.057 (95% CI, 1.083–15.201); *p* = 0.038) groups. However, the incidence of individual *GSTM1*-null or combined *GSTM1*/*GSTT1* double-null genotypes did not vary significantly between the groups. Our collective findings support the utility of the *GSTT1*-null genotype as a useful biomarker for liver disease progression in Brazilian patients with chronic hepatitis C.

## 1. Introduction

With a reported 905,677 new cases and 830,180 deaths in 2020, liver cancer is predicted to be the sixth leading cause of cancer and third highest cause of cancer-associated mortality worldwide, presenting a major global health problem [1]. Hepatocellular carcinoma (HCC) is the most common form of liver malignancy and accounts for ~90% of cases [2]. In most cases, HCC develops in the context of chronic liver inflammation and cirrhotic transformation, due to viral hepatitis, alcohol abuse, or nonalcoholic fatty liver disease [3]. Cirrhosis of any etiology is a main risk factor for development of HCC. Among cirrhotic patients, those with hepatitis C virus (HCV) infection have the highest risk of developing HCC, even after viral eradication [4]. In Brazil, 10,902 deaths were attributed to HCC in 2019 [5], the major risk factor being chronic HCV infection, which accounted for ~65% of the cases [6].

According to the World Health Organization (WHO), an estimated 71 million people worldwide have chronic HCV infection, resulting in 399,000 deaths, mainly attributable to cirrhosis and HCC [7]. Despite tremendous improvements in HCV treatment in recent years, many patients remain at risk of disease progression to cirrhosis and HCC [8,9]. Disease progression is influenced by viral, genetic, and epigenetic factors [10]. Numerous candidate-gene studies have reported associations between genetic polymorphisms and the presence of HCC [11,12,13,14,15]. Since oxidative stress is a critical factor in the pathogenesis of chronic hepatitis C [16], genetically induced differences in antioxidant mechanisms may modulate the natural history of the disease.

Glutathione S-transferases (GST) consist of an essential enzymatic system implicated in the cellular mechanism of detoxification that protects cells against oxidative stress. At least seven different classes of GSTs are highly expressed in the mammalian liver. GSTM1 and GSTT1 activities are modulated by genetic polymorphisms, and these isoforms are the most extensively studied in relation to risk of environment-related human diseases. GST Mu class (*GSTM1*) is located on the short arm of chromosome 1 (1p13.3) and GST Theta class (*GSTT1*) is on the long arm of chromosome 22 (22q11.23) [17,18]. Both genes have a null variant allele (partially deleted), which results in abrogation of catalytic activity and consequent enhancement of oxidative stress and cytotoxic damage in the liver. Individuals carrying homozygous deletions in these genes are considered to be at higher risk for malignancy, owing to their reduced capacity to detoxify potential carcinogens [19]. Previous studies support an association between *GSTM1* and *GSTT1* polymorphisms and risk of HCC (including both viral and non-viral etiologies). However, the majority of these studies were performed in Asian populations [20,21,22].

Limited published data are available on the influence of GST polymorphisms in the late stages of HCV infection, specifically, development of cirrhosis and HCC [23,24,25], and no studies have been conducted on the Brazilian population to date. In this study, we determined the distribution of *GSTM1* and *GSTT1* polymorphisms in patients with chronic HCV grouped according to stage of liver disease (chronic hepatitis, cirrhosis, and HCC), with a view to investigating their potential involvement in risk of disease progression in Brazilian patients with chronic hepatitis C infection.

## 2. Materials and Methods

### 2.1. Patients

Blood samples were collected from 190 patients with HCV chronic infection at different stages of liver disease, including 47 with chronic hepatitis, 83 with cirrhosis (without HCC), and 60 with HCC, referred to the Clementino Fraga Filho University Hospital (HUCFF) in Rio de Janeiro, Brazil, from 2012 to 2015. Cases with viral co-infections (hepatitis B virus, hepatitis A virus, hepatitis E virus, human immunodeficiency virus), alcoholics, and those with other etiologies for chronic liver diseases were excluded. Diagnosis of liver cirrhosis was based on liver biopsy or presence of clinical and laboratory features of portal hypertension at ultrasound or upper endoscopy. HCC diagnosis was made based on at least one positive image on magnetic resonance imaging or computed tomography. Demographic, laboratory, and clinical data were obtained from the medical records of patients. The research protocol was approved by the Ethics Committee of the hospital (agreement number 139/10) and informed consent was obtained from all patients.

### 2.2. Genotyping of GSTM1 and GSTT1 Polymorphisms

**Figure 1:**
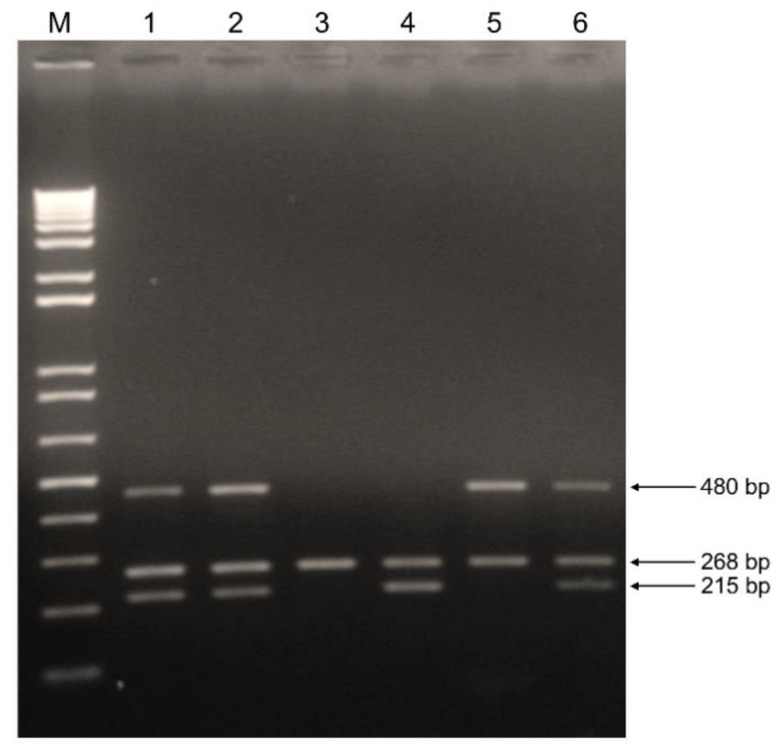
Genotyping of *GSTM1* and *GSTT1* by multiplex PCR. Lane M: 1 Kb Plus DNA Ladder. Lanes 1, 2 and 6: positive *GSTM1* and *GSTT1* genotypes. Lane 3: null *GSTM1* and *GSTT1* genotypes (absence of 215 and 480 bp fragments). Lane 4: null *GSTT1* genotype (absence of 480 bp fragment). Lane 5: null *GSTM1* genotype (absence of 215 bp fragment). The β-globin internal control is detected as 268 bp fragment.

Genomic DNA was extracted from 200 µL aliquots of serum samples using the High Pure Viral Nucleic Acid kit (Roche Diagnostics, Basel, Switzerland) according to the manufacturer’s instructions. *GSTM1* and *GSTT1* polymorphisms were detected based on multiplex polymerase chain reaction (PCR) using β-globin as an internal control according to the report of Unal et al. [26]. Briefly, a 50 μL multiplex reaction mixture, containing 4 μL nucleic acid template, 2U Platinum *Taq* DNA Polymerase, 10 pmol forward and reverse primers and supplied reagents (Invitrogen, Waltham, MA, USA), was used for PCR. Amplification conditions comprised 35 cycles of denaturation at 94 °C for 1 min, annealing at 62 °C for 1 min, and extension at 72 °C for 1 min, resulting in a fragment of 480 bp for *GSTT1*, 215 bp for *GSTM1*, and 268 bp for β-globin. PCR reactions were performed on an ABI 2720 Thermal Cycler (Applied Biosystems, Waltham, MA, USA). A negative control containing water instead of DNA was included in all amplification reactions. PCR products were analyzed on a 2% agarose gel and stained with ethidium bromide (Figure 1). The subjects were classified as either positive (when at least one copy of the gene was present) or null genotypes. Heterozygous and homozygous individuals for *GSTM1* or *GSTT1* have been reported to present similar enzyme expression levels [27] and were considered together for statistical analysis.

### 2.3. Statistical Analysis

Statistical analysis using SPSS 20.0 software (IBM, Armonk, NY, USA) was performed to calculate odds ratio (OR) and 95% confidence intervals (CI) to assess the relative disease risk conferred by a specific genotype. Polymorphism distribution and categorical variables of patient data were compared using Pearson’s χ^2^ or Fisher’s exact test, as appropriate. Mann–Whitney and Student’s t-test were used to compare quantitative variables between groups. *p*-values < 0.05 were considered statistically significant.

## 3. Results

### 3.1. Characteristics of Patients

Table 1 shows the demographic and biochemical characteristics of 190 HCV-infected patients classified as chronic hepatitis, cirrhosis (without HCC), and HCC carriers. The mean age of HCC patients was significantly higher than that of patients with chronic hepatitis (*p* < 0.001) and cirrhosis (*p* = 0.006). The group with chronic hepatitis had a lower proportion of males, compared to the HCC (*p* = 0.015) group. No statistically significant differences were detected between HCC and cirrhosis groups in relation to sex and levels of alanine aminotransferase (ALT), aspartate aminotransferase (AST), alkaline phosphatase (ALP), gamma-glutamyl transpeptidase (GGT), serum albumin (ALB), and total bilirubin (T.BIL). However, the group with chronic hepatitis had significantly lower ALT, AST, ALP, GGT, and T.BIL values than the HCC group (*p* < 0.05 for all observations). Moreover, alpha-fetoprotein (AFP) levels were significantly higher in HCC patients than those with cirrhosis and chronic hepatitis (*p* < 0.01 for both). Data on HCV genotypes were available for 131 (69%) patients. The frequencies of genotypes 1a, 1b, and 3a in the total group were 45%, 41% and 14%, respectively. No significant differences were observed between HCC, cirrhosis, and chronic hepatitis groups in relation to HCV genotype (Table 1).

### 3.2. GSTM1 and GSTT1 Polymorphisms and Risk for Cirrhosis and HCC

The distribution frequencies of GSTM1 and GSTT1-null genotypes between patients with cirrhosis and chronic hepatitis are presented in Table 2. The incidence of the GSTT1-null genotype was significantly increased in patients with cirrhosis relative to the chronic hepatitis group (odds ratio, OR, 3.778 (95% confidence interval, CI, 1.045–13.659); *p* = 0.043). No significant differences were observed for individual GSTM1-null and combined GSTM1/GSTT1 double-null genotypes (Table 2).

Table 3 shows the distribution of GSTM1 and GSTT1-null genotypes between patients with HCC and chronic hepatitis. A markedly higher frequency of GSTT1-null genotype was observed in HCC than chronic hepatitis patients (OR, 4.057 [95% CI, 1.083–15.201]; *p* = 0.038). We observed no significant differences with regard to other genotypes, although GSTM1/GSTT1 double-null genotype was more frequently detected in HCC patients than in those with chronic hepatitis (10% and 2.1%, respectively).

Analysis of liver biochemistry tests according to *GSTM1* and *GSTT1* genotypes is shown in Table 4. ALT, AST, ALP, GGT, ALB and T.BIL levels were not significantly different between genotypes (*p* > 0.05). Similarly, no significant differences in levels among the genotypes were detectable when patients from chronic hepatitis, cirrhosis, and HCC groups were analyzed individually (data not shown).

## 4. Discussion

Although several studies indicate that GST genotypes are associated with HCC (including both viral and non-viral etiologies), variable results according to ethnicity have been obtained. In two recent meta-analyses [20,22], increased risk of HCC was observed in Asians with *GSTM1* and *GSTT1* single-null and *GSTM1*/*GSTT1* double-null genotypes, while no significant differences were detected among Caucasians or Africans. These differences may be attributed to diverse living surroundings and inherited backgrounds of the studied populations.

The Brazilian population has a highly admixed genetic background [28], and candidate gene-based association studies exploring the relationship between genetic polymorphisms and HCC are rare in Brazil. Since oxidative stress plays an important role in the pathogenesis of chronic hepatitis C [16], genetically induced differences in antioxidant mechanisms may influence disease severity. To our knowledge, the present study is the first to investigate the association of *GSTM1* and *GSTT1* polymorphisms with advanced liver disease induced by HCV infection in Brazilian patients. Our results suggest that the *GSTT1*-null genotype increases the risk of cirrhosis and HCC, since the frequency of this genotype was significantly higher in patients with cirrhosis (without HCC) and HCC relative to those with chronic hepatitis. Conversely, the frequency of the *GSTM1*-null genotype was similar in all groups and not associated with development of advanced liver disease in our study population. Our findings are consistent with a previous Brazilian study focusing on the association between GST polymorphisms and the risk of developing HCC (including both viral and non-viral etiologies), which showed that the *GSTT1*-null, but not *GSTM1*-null genotype, was associated with HCC [29]. Likewise, significantly higher frequency of the *GSTT1*-null genotype was observed in Spanish patients with HCV-induced liver cirrhosis compared with healthy control subjects [24]. Interestingly, GSTT1 has glutathione peroxidase activity and may be directly involved in detoxification of reactive species produced as a consequence of ongoing HCV infection of the liver [24,30]. Notably, two previous studies evaluating the association of *GSTM1* and *GSTT1* polymorphisms with progression of liver disease in Egyptian [23] and Taiwanese [25] patients with chronic HCV showed considerably increased risk of advanced fibrosis in individuals with combined *GSTM1*/*GSTT1* double-null genotype. Here, the *GSTM1*/*GSTT1* double-null genotype was more frequently detected in patients with HCC than those with chronic hepatitis (10% vs. 2.1%, respectively) but this difference was not statistically significant. Notably, the *GSTT1*-null genotype could induce a subtle but functionally significant deficit in hepatic antioxidant and reactive oxygen species (ROS)-inactivating mechanisms. The simultaneous lack of GSTM1 enzymatic function may reinforce this deficit, and consequently, more ROS and electrophilic compounds generated during HCV infection are present in individuals devoid of both GST enzymes [16,24]. The resulting deficit in antioxidant mechanisms may facilitate the amplification of the inflammatory reaction during chronic hepatitis C infection, increasing the risk of disease progression to cirrhosis and HCC.

We further investigated whether *GSTM1* and *GSTT1*-null genotypes indirectly contribute to the levels of liver biochemistry tests (ALT, AST, ALP, GGT, ALB, T.BIL) in chronic HCV infection cases. No significant differences in the levels were observed between positive and null genotypes in the whole study population or upon individual analysis of patients with chronic hepatitis, cirrhosis, and HCC. To our knowledge, no previous studies have focused on the influence of *GSTM1* and *GSTT1* polymorphisms on liver biochemistry tests in chronic HCV infection. Potential limitations of this study include small sample size and older age of the patients with HCC.

Predisposition to disease severity based on genetic risk has been extensively explored as a path to personalized medicine. The ability of polymorphic traits, alone, to refine individual prognosis has been considered insufficient for introduction into clinical practice as predictive markers [11,14]. However, future integration of several panels of genes may define a “genomic risk prediction” and improve the existing risk-assessment models for HCC.

In conclusion, the *GSTT1*-null genotype was significantly more frequent in the most severe presentations of HCV-related liver disease among Brazilian patients, supporting its potential as a molecular marker for assessing risk of progression of chronic hepatitis C to cirrhosis and HCC. However, further large-scale population-based studies are warranted to validate these findings.

## Figures and Tables

**Table 1:** Demographic and laboratory data on HCV-infected patients.

				*p*-Value		
Characteristics	Chronic Hepatitis	Cirrhosis	HCC	HCC	HCC	Cirrhosis
	(*n* = 47)	(*n* = 83)	(*n* = 60)	vs.	vs.	vs.
				Chronic Hepatitis	Cirrhosis	Chronic Hepatitis
Age (years, mean ± SD) ^a^	57 ± 11	59 ± 9	64 ± 9	<0.001	0.006	0.48
Male (%)	14 (30)	42 (51)	32 (53)	0.015	0.74	0.99
ALT (U/L, median (IQR))	59 (41)	74 (75)	82 (30)	0.029	0.88	0.067
AST (U/L, median (IQR))	43 (40)	70 (77)	91 (62)	0.001	0.30	0.008
ALP (U/L, median (IQR))	80 (35)	110 (58)	123 (134)	<0.01	0.18	<0.01
GGT (U/L, median (IQR))	68 (46)	101 (146)	113 (155)	0.002	0.32	0.012
ALB (g/dL, median (IQR))	3.8 (0.4)	3.6 (1.0)	3.6 (1.0)	0.70	0.74	0.018
T.BIL (mg/dL, median (IQR))	0.7 (0.3)	0.8 (0.8)	0.9 (0.6)	0.005	0.40	0.016
AFP (ng/mL, median (IQR))	6.9 ± (7.4)	6.0 (15)	31 (248)	<0.01	<0.01	0.52
HCV genotype (%)						
1a	10 (21)	31 (37)	18 (30)	0.378	0.378	0.076
1b	15 (32)	20 (24)	19 (32)	1.000	0.345	0.411
3a	4 (9)	5 (6)	9 (15)	0.380	0.091	0.722
ND	18 (38)	27 (33)	14 (23)	-	-	-

^a^ One-way ANOVA, Bonferroni post hoc test. ALT, alanine aminotransferase; AST, aspartate aminotransferase; ALP, alkaline phosphatase; GGT, gamma-glutamyl transpeptidase; ALB, serum albumin; T.BIL, total bilirubin; AFP, alpha-fetoprotein; SD, standard deviation; IQR, interquartile range; ND, not determined.

**Table 2:** Distribution of GSTM1 and GSTT1-null genotypes according to presence/absence of cirrhosis.

Genotype	Chronic Hepatitis (*n* = 47)	Cirrhosis (*n* = 83)	*p*-Value	OR	95% CI
*GSTM1*-null (%)	22 (46.8%)	34 (41%)	0.518	1.268	0.617–2.618
*GSTT1*-null (%)	3 (6.4%)	17 (20.5%)	0.043	3.778	1.045–13.659
*GSTM1*-null/*GSTT1*-null (%)	1 (2.1%)	3 (3.6%)	0.567	1.522	0.193–20.131

OR, odds ratio; 95% CI, 95% confidence interval.

**Table 3:** Distribution of *GSTM1* and *GSTT1*-null genotypes according to presence/absence of HCC.

Genotype	Chronic Hepatitis (*n* = 47)	HCC (*n* = 60)	*p*-Value	OR	95% CI
*GSTM1*-null (%)	22 (46.8%)	28 (46.7%)	0.98	1.006	0.468–2.162
*GSTT1*-null (%)	3 (6.4%)	13 (21.7%)	0.038	4.057	1.083–15.201
*GSTM1*-null/*GSTT1*-null (%)	1 (2.1%)	6 (10%)	0.127	5.520	0.617–49.393

OR, odds ratio; 95% CI, 95% confidence interval.

**Table 4:** Levels of liver biochemistry tests according to *GSTM1* and *GSTT1* genotypes.

Variables	*GSTM1* Null	*GSTM1* Positive	*GSTT1* Null	*GSTT1* Positive	*p*-Value	
					*GSTM1* Null vs. *GSTM1* Positive	*GSTT1* Null vs. *GSTT1* Positive
ALT (U/L, median (IQR))	78 (67)	71 (45)	77 (42)	71 (59)	0.283	0.907
AST (U/L, median (IQR))	70 (93)	69 (73)	85 (69)	68 (78)	0.279	0.748
ALP (U/L, median (IQR))	97 (54)	107 (79)	116 (72)	103 (76)	0.074	0.206
GGT (U/L, median (IQR))	85 (181)	102 (130)	89 (113)	94 (141)	0.692	0.937
ALB (g/dL, median (IQR))	4.0 (1.0)	4.0 (1.0)	3.0 (1.2)	4.0 (0.8)	0.913	0.173
T.BIL (mg/dL, median (IQR))	1.0 (0.9)	1.0 (0.5)	1.0 (0.9)	1.0 (0.7)	0.420	0.839

ALT, alanine aminotransferase; AST, aspartate aminotransferase; ALP, alkaline phosphatase; GGT, gamma-glutamyl transpeptidase; ALB, serum albumin; T.BIL, total bilirubin; IQR, interquartile range.
